# HeteroGenome: database of genome periodicity

**DOI:** 10.1093/database/bau040

**Published:** 2014-05-24

**Authors:** Maria Chaley, Vladimir Kutyrkin, Gayane Tulbasheva, Elena Teplukhina, Nafisa Nazipova

**Affiliations:** ^1^Laboratory of Bioinformatics, Institute of Mathematical Problems of Biology, Russian Academy of Sciences, Institutskaya st. 4, 142290 Pushchino, Russia and ^2^Department of Computational Mathematics and Mathematical Physics, Moscow State Technical University n.a. N.E. Bauman, the 2nd Baumanskaya st., 5, 105005 Moscow, Russia

## Abstract

We present the first release of the HeteroGenome database collecting latent periodicity regions in genomes. Tandem repeats and highly divergent tandem repeats along with the regions of a new type of periodicity, known as profile periodicity, have been collected for the genomes of *Saccharomyces cerevisiae*, *Arabidopsis thaliana*, *Caenorhabditis elegans* and *Drosophila melanogaster*. We obtained data with the aid of a spectral-statistical approach to search for reliable latent periodicity regions (with periods up to 2000 bp) in DNA sequences. The original two-level mode of data presentation (a broad view of the region of latent periodicity and a second level indicating conservative fragments of its structure) was further developed to enable us to obtain the estimate, without redundancy, that latent periodicity regions make up ∼10% of the analyzed genomes. Analysis of the quantitative and qualitative content of located periodicity regions on all chromosomes of the analyzed organisms revealed dominant characteristic types of periodicity in the genomes. The pattern of density distribution of latent periodicity regions on chromosome unambiguously characterizes each chromosome in genome.

**Database URL:** http://www.jcbi.ru/lp_baze/

## Introduction

### Periodicity in genomes

Periodicity regions of tandem repeats (arrays of sequentially repeated copies of original DNA sequence fragments, or patterns) in genomes have long been of interest for two main reasons: to understand the molecular mechanisms of the origin and evolution of the repeats and their functional role in genomes, and to determine potential new markers for population and evolutionary genetics. Alterations in pattern copies (substitution of original nucleotides, insertions and deletions of nucleotides) lead to the formation of approximate tandem repeats. Repeats whose alterations are limited to substitutions of nucleotide bases (bp) are commonly called fuzzy tandem repeats. Approximate tandem repeats (including fuzzy repeats) are regions of latent periodicity in a genome.

The most studied groups of tandem repeats in genomes are microsatellites (patterns of ∼10 bp) and minisatellites (patterns of ∼100 bp) because of their use as genetic markers in forensics, parentage assessment, positional cloning and population and evolutionary genetics (1).

Microsatellite tandem repeats are generally believed to arise by replication slippage (2). In contrast to microsatellites, DNA recombination-like processes that involve unequal crossover or gene conversion display mutation mechanisms in the larger minisatellite sequences (3). Tandem repeats can arise by consecutive gene duplication as a result of homologous chromosome recombination during meiosis (4).

Numerous tandem repeats are situated in centromeric and telomeric regions of chromosomes (5). Tandem repeats have been found at fragile chromosome sites (6, 7). In some cases, the expansion of triplet microsatellites at fragile sites caused human mental retardation (8). Human neurological disorders may also be induced by ‘dynamic’ mutations (reduction or increase in the pattern copy number of a tandem repeat) in both coding regions and noncoding regions; such mutations are not limited to occurring with triplet microsatellites (9–11). Tandem repeats situated outside the boundaries of coding regions influence the expression of genes and the processes of transcription and translation (12).

### Periodicity software and databases

A number of computer programs have been developed to search for perfect or nearly perfect tandem repeats, including the programs TRF (13), ACMES (14), MREPATT (15), STRING (16), mreps (17) and ATRHunter (18). These programs are based on various algorithms, and whether their results agree is dependent on the period length, copy number and divergence of a repeat.

Over the past decade, programs have also been developed to search for increasingly divergent tandem repeats with the aim of studying their evolutionary aspects, including TandemSWAN (19), IMEX (20), TRStalker (21), and a program based on a model of evolutive tandem repeats (22). Whereas perfect (or nearly perfect) tandem repeats vary by copy number and are dynamic because of strand slippage during DNA replication, highly divergent repeats are relatively stable structural genomic elements, but their functional role has so far been insufficiently studied.

Search results for tandem repeats are collected in various resources such as the TRedD (23) database of tandem repeats, determined in the human genome with the aid of an algorithm described in (22). The best-known database, TRDB (24), contains tandem repeats revealed via the Tandem Repeats Finding (TRF) method (13) in entire sequenced genomes of eukaryotes (including human) and prokaryotes. TRbase (25) connects tandem repeats found in the human genome using the TRF method (13) with gene location on the chromosomes, and in particular highlights those genes whose disorders result in genetic diseases.

It should be noted that proposed heuristic algorithms revealing highly divergent approximate tandem repeats do not resolve the problem of the reliability of results. To ensure that an approximate tandem repeat has been found, additional filtration of the results is usually performed. For example, in the program based on a model of evolutive tandem repeats (22), the level of pattern copy divergence in a repeat is limited to a maximum of ∼30%. However, this value exceeds the divergence level of 20% required in a probability model for the TRF method (13). Moreover, redundancy of the TRF results (different patterns in the same tandem repeat may be proposed) (19) and instability of the results (for example, shifting of 1–3 bp along the same DNA sequence can lead to different pattern estimates) have been reported (26).

Along with the methods mentioned above, there is also a statistical method (27, 28) that is known as information decomposition method (ID-method). The authors of the ID-method have created the MMsat database (29) and web-server LEPSCAN (30) for searching latent periodicity regions with period length ranging from 2 to 20 bp. Practical utility of the MMsat database is restricted by the latent micro- and minisatellites found out in the GenBank with the help of ID-method.

To estimate a period length of latent periodicity in DNA sequence, the authors of the ID-method introduce Z-statistics, whose properties are based on the empiric samples. The foundation of such Z-statistics is a standard information statistics (31) applicable, as shown by Kullback (31), only for revealing heterogeneity in analyzed sequence. So, Z-statistics can be used only for the same goals. Hence, the empiric properties of Z-statistics do not guaranty the existence of latent periodicity with unknown structure in analyzed sequence. As a consequence, it is only possible to obtain the incorrect estimates for period of latent periodicity, relying on such properties. Further, we will compare our approach with the ID-method in more detail.

In our work, an original spectral-statistical approach (SS-approach) (32) was applied to search for reliable latent periodicity regions in the genomes of *Saccharomyces cerevisiae*, *Arabidopsis thaliana*, *Caenorhabditis elegans* and *Drosophila melanogaster*. As previously shown (26, 32), such an approach prevents nonuniqueness when resolving latent periodicity structure in approximate tandem repeats and optimizes estimation of the periodicity pattern size.

The SS-approach reveals extremely divergent tandem repeats (pattern copy divergence level ∼50%) and regions of a new type of periodicity known as profile periodicity (33, 34). Methods commonly applied to locate approximate tandem repeats cannot be used to locate profile periodicity.

### Latent periodicity and heterogeneity

Notion of latent profile periodicity or latent profility (32) expands on the notion of approximate tandem repeat (13), which was earlier used for recognizing latent periodicity in DNA sequences. Perfect tandem repeat is a textual string that consists of sequential copies of its substring called a periodicity pattern. In approximate tandem repeat, a small number (no more than 30%) of characters in pattern copies are distorted by indels and nucleotide point mutations. If latent profile periodicity exists in DNA sequence, distortion of the characters in each position of pattern copies occurs in accordance with the corresponding probability distribution.

In a model of latent profility, a DNA sequence (textual string) is considered as a realization of a special random periodic string called a profile string (33, 34). This string, consisting of independent random characters, represents a perfect tandem repeat of a random string, a so-called random periodicity pattern. This pattern of latent profility consists of independent random characters with a corresponding probability distribution for letters from the DNA alphabet. Exploring latent profility is beyond the scope of the present article. An example of a sequence with latent profility will be discussed further.

SS-approach, used when analyzing model organisms, allowed to spot the periodicity of two types—approximate tandem repeats (13) and latent profility (32–34). Moreover, there are a number of the repeat-like regions, also meeting a criterion of significant heterogeneity in accordance with the SS-approach used for revealing latent periodicity. General description of the approach will be done in the next section. The approach uses χ^2^-statistics for testing homogeneity in DNA sequence at significance level characteristic for approximate tandem repeats whose sequences are obviously heterogenic ones. However, a significant heterogeneity is a necessary, but insufficient, condition for determining latent periodicity of the types mentioned above.

As the results of latent periodicity search in automatic mode are undoubtedly heterogenic sequences and some additional analysis to verify their periodic structure is needed, further, for brevity, these results are frequently referred to as heterogeneities. So, the name of the database HeteroGenome reflects this particularity of the data collected. There are additional tools in the HeteroGenome for revealing latent periodicity, which will be further described in ‘Additional sequence analysis’ section.

This article presents the first release of the HeteroGenome database, which collects latent periodicity regions (with periods up to 2000 bp) revealed by the SS-approach (32) in the genomes of various organisms. Because of nonredundancy of the results of the approach, for each genome under consideration we can estimate its coverage as a percentage of latent periodicity regions, and with the aid of a special parameter, which is described in the next section, we can analyze the conservation of periodic structure in the regions. Such analysis enables dominant characteristic types of periodicity to be located in each of the genomes. Therefore, HeteroGenome may be useful for both functional genomics and evolutionary genomics in searching for tandem repeats characteristic of the genome and for further research into the phenomenon of latent periodicity in DNA sequences. The database has a user-friendly interface and options for additional data analysis and allows query results to be downloaded. HeteroGenome can be freely accessed at http://www.jcbi.ru/lp_baze/.

## Materials and methods

### Genomic data

The first release of the HeteroGenome database was not aimed at collecting a large amount of data from a large number of organisms. First and foremost, the data should serve to mining of scale and character of latent periodicity phenomenon in the genome of living organisms. Therefore, in our work, an emphasis was applied to quantitative and qualitative analysis of the regions with latent periodicity revealed by the original SS-approach (26, 32), which is described in the next section. For this purpose, the four genomes of well-studied model organisms (35)—*S. cerevisiae*, *A. thaliana*, *C. elegans* and *D. melanogaster*—were selected. The genomes of these organisms were among the first to be sequenced and, from them, moderately accurate and well-annotated data were obtained. Some representative eukaryotes, ranging from unicellular (yeast) to multicellular organisms of the plant (*Arabidopsis*) and animal (*Drosophila*, nematode) kingdoms, also facilitate an overview of latent periodicity in the genome. The original DNA sequences of the entire genomes of *S. cerevisiae*, *A. thaliana*, *C. elegans* and *D. melanogaster* were taken from ftp://ftp.ncbi.nih.gov/genomes/. Data on chromosome contigs are shown in Table 1.

**Table 1.** Analyzed genomes of model organisms

Species	Chromosomes	GenBank identifiers	
*S. cerevisiae*	I	NC_001133.7	GI:144228165
	II	NC_001134.7	GI:50593115
	III	NC_001135.4	GI:85666111
	IV	NC_001136.8	GI:93117368
	V	NC_001137.2	GI:7276232
	VI	NC_001138.4	GI:42742172
	VII	NC_001139.8	GI:162949218
	VIII	NC_001140.5	GI:82795252
	IX	NC_001141.1	GI:6322016
	X	NC_001142.7	GI:116006492
	XI	NC_001143.7	GI:83722562
	XII	NC_001144.4	GI:85666119
	XIII	NC_001145.2	GI:44829554
	XIV	NC_001146.6	GI:117937805
	XV	NC_001147.5	GI:84626310
	XVI	NC_001148.3	GI:50593503
	MT	NC_001224.1	GI:6226515
*A. thaliana*	I	NC_003070.9	GI:240254421
	II	NC_003071.7	GI:240254678
	III	NC_003074.8	GI:240255695
	IV	NC_003075.7	GI:240256243
	V	NC_003076.8	GI:240256493
*C. elegans*	I	NC_003279.4	GI:86561680
	II	NC_003280.4	GI:86562519
	III	NC_003281.5	GI:86563600
	IV	NC_003282.3	GI:72185816
	V	NC_003283.5	GI:86564547
	X	NC_003284.5	GI:86565306
*D. melanogaster*	2L	NT_033779.4	GI:116010444
	2R	NT_033778.3	GI:116010442
	3L	NT_037436.3	GI:116010443
	3R	NT_033777.2	GI:56411841
	4	NC_004353.3	GI:116010290
	X	NC_004354.3	GI:116010291

### SS-approach to revealing latent periodicity in DNA

We used an original SS-approach (26, 32) to reveal latent periodicity in DNA. The SS-approach reveals latent periodicity by detecting significant heterogeneity at the test-periods of an analyzed nucleotide sequence. At each test-period *λ*, the analyzed sequence is divided into substrings of length *λ* (the final substring may have a smaller length). If *n* is the length of the analyzed sequence, then *R*_λ_=n/λ is its test-exponent for the test-period *λ*. This division by substrings allows us to calculate the frequency of occurrence 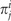 of the *i*th letter of a nucleotide sequence alphabet in the *j*th positions of a test-period. Matrix 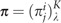 is the sample *λ*-profile matrix for the analyzed sequence, where *K* is the size of the nucleotide sequence alphabet. Frequency occurrence *p*^*i*^ of the *i*th letter in the analyzed sequence is determined by the matrix 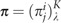:
(1)
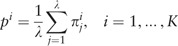



For checking sequence heterogeneity at test-period *λ*, normalized Pearson χ²-statistics (32) are applied:
(2)
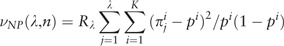



Because the search for tandem repeats is conducted in nucleotide databases that are large in volume, to verify heterogeneity in DNA sequences a significance level (type-I error) of α = 10^−6^ was chosen. At fixed test-period *λ*, the level corresponds to a critical value of χ_*crit*_²(α, *N*) with *N* = (*K*−1)(λ−1) degrees of freedom. Therefore, if ν_*NP*_(λ, *n*) for the analyzed sequence of length *n* at the test-period *λ* meets the condition
(3)
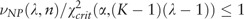

then homogeneity of the sequence is assumed at test-period *λ*; otherwise, the sequence is considered heterogenic. Thus, as a spectral characteristic of the analyzed nucleotide sequence, the following function **H** is used:
(4)
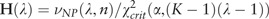

where 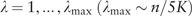.

A plot of the function **H** (**H**-spectrum), which is the *spectrum of heterogeneity manifestation* in the analyzed sequence, demonstrates clearly the manifestation of significant sequence heterogeneities at those test-periods where **H**(λ) > 1. These test-periods form a *spectrum of heterogeneity structure* for the sequence, which is further analyzed with the aid of an additional spectral-statistical characteristic.

At every test-period *λ* of the analyzed sequence according to the sample *λ*-profile matrix 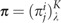, an additional parameter is calculated,
(5)
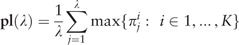

which is the *character preservation level* at test-period *λ*.

**Figure 1.**
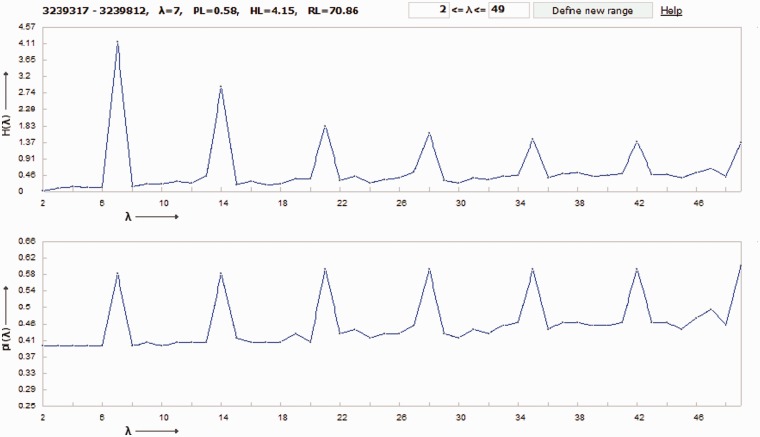
Spectral-statistical characteristics of DNA sequence in the HeteroGenome database. For a sequence on chromosome IV (3239317–3239812 bp) of *A. thaliana*, spectral-statistical characteristics reveal a periodicity pattern size of 7 bp. Spectrum of heterogeneity manifestation [**H**-spectrum, see Equation (4)] is shown above. Spectrum of character preservation level [**pl**-spectrum, see Equation (5)] is shown below. If the spectra are too long, they may be viewed in parts. The button ‘Define new range’ is used to specify the desired part.

Therefore, for the considered sequence, the spectrum of heterogeneity structure is analyzed with the spectrum of character preservation level (**pl**-spectrum). The test-period from the spectrum of heterogeneity structure, indicated by the first maximal value in the spectrum of character preservation level, is considered to be an estimate of periodicity pattern size for region periodicity (see Figure 1). This maximal value in the **pl**-spectrum may be interpreted as an average index of preservation for copies of the estimated periodicity pattern.

The problem of reliability in locating approximate tandem repeats under the condition of small sample size (when the number of pattern copies in the repeat is sufficiently small) in the case of the SS-approach is resolved using the stochastic model of heterogeneity manifestation in textual strings (32). This model allows us to use additional statistical tests to check a hypothesis of heterogeneity presence in a DNA sequence.

From an algorithmic point of view, determining latent periodicity is difficult if a period is *a priori* unknown. We therefore searched for latent periodicity by identifying significant heterogeneities (at level α = 10^−6^) in overlapping windows of various sizes, in analysis of multiple-scanned DNA sequences with variable steps. The initial window size was 30 bp. The size of each subsequent window was double that of the previous window, to a maximum of 4000 bp. Thus, the broad strategy used by the program, applying the SS-approach (32) to search for latent periodicity regions, resembled a ‘shotgun strategy’ for sequencing the genomes (36). Under this strategy, relatively short and overlapping DNA fragments were first sequenced and then assembled by the program into longer regions. Thus, initial data on regions of significant heterogeneity found in the genomes of the organisms passed through a number of specific procedures to enable additional processing and obtain well-defined borders for the located regions of heterogeneity.

### Particularities of SS-approach

Let us compare the SS-approach used in the present work with the already mentioned ID-method (27, 28) on the basis of which MMsat database (29) and web-server LEPSCAN (30) have been created. To estimate a period of latent periodicity the ID-method exploits Z-statistics relying on information statistics, earlier introduced by Kullback (31). As it was mentioned above, the information statistics along with Z-statistics can be used only for revealing heterogeneity in analyzed sequence.

Pearson statistics [see Equation (2)] is used at preliminary stage in the SS-approach to reveal heterogeneity in an analyzed sequence. Heterogeneity can also be manifested at the shorter test-periods than existing latent period. To estimate length for period of latent periodicity, a spectrum is introduced for another original statistics **pl**[see Equation (5)] called ‘character preservation level at test-period’. This statistics, contrary to Pearson statistics (32, 37), information statistics (31) and Z-statistics (28), continues to be representative up to the test-period equaled to the one halve of analyzed sequence length because in the positions of each test-period it checks up matching (mismatching) the characters independently of their kind. Hence, such a test can be considered as binomial schema. So, if the high level (∼0.5 and more) of character preservation is determined for analyzed DNA sequence, its proximity to divergent tandem repeat is naturally supposed. Such a supposition is used as the main criterion in selecting the sequences for the HeteroGenome database. It should be additionally mentioned that if the high level of character preservation is revealed at the test-period λ, the relative λ-profile matrix can be considered as representative for the test-period, which was studied in (32). Consequently, Pearson statistics and the original statistics of character preservation level at test-period are complementary statistics. This allows for the HeteroGenome database to select the sequences that are proximal to the approximate tandem repeats. Special visual presentation of a sequence in the HeteroGenome helps to define the boarders of an approximate tandem repeat more exactly.

**Figure 2.**
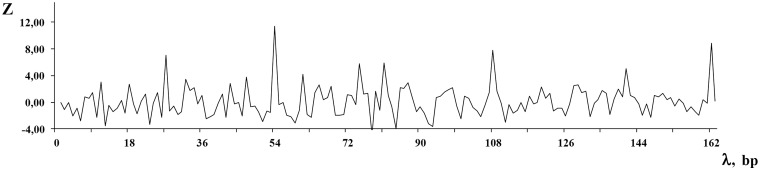
Spectrum of Z-statistics is shown for the DNA sequence from the MMsat database (AAU92263.1). The considered sequence is a fragment of an original sequence from GenBank (AAU92263, indices: 61–388 bp).

**Figure 3.**
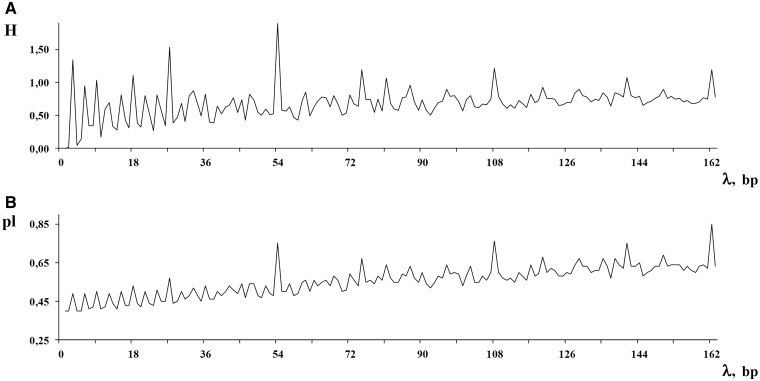
The spectra of the SS-approach for DNA sequence from the MMsat database (AAU92263.1). (**A**) Spectrum of heterogeneity manifestation [see Equation (4)]. (**B**) Spectrum of character preservation level [see Equation (5)].

**Figure 4.**
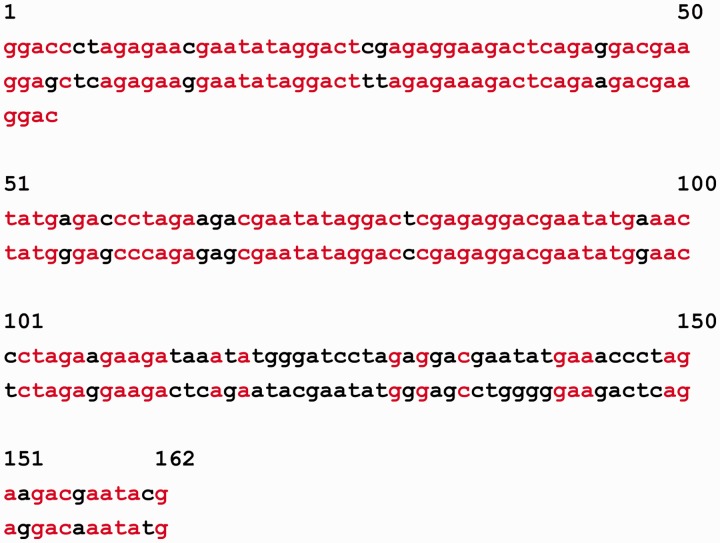
Alignment of DNA sequence from the MMsat database (AAU92263.1) is shown according to the estimate of 162 bp for latent period, which was obtained with the help of SS-approach. Matching characters in the positions of the tandem repeat of the two copies are shown in red.

Let us give an example of DNA sequence from the MMsat database (29), which was analyzed with the help of SS-approach. Spectrum of Z-statistics obtained from the MMsat for this sequence is shown in the Figure 2. Based on the maximal peak in Z-statistics spectrum, a conclusion was done about existing in the sequence latent periodicity with period of 54 bp and with unknown structure. In considering the **pl** spectrum (see Figure 3B), one can see that maximal value of character preservation level for this sequence is achieved at the test-period λ = 162 bp where 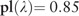. Therefore, the analyzed DNA sequence is an approximate tandem repeat (see Figure 4). This example demonstrates that in the case of the high values of the **pl** spectrum the upper limit for permitted test-periods in the **H** spectrum can be significantly enlarged as it was done here—up to a halve of sequence length (see Figure 3A).

It should be noted that in the MMsat database (29) a period estimate is limited by the value of 100 bp. Employment of the character preservation level statistics in the HeteroGenome database allows extending this bound up to 2000 bp and even more.

It should be also mentioned that spectrum of Z-statistics (28) is nonreproducible for other researchers. This is owing to the fact that the Monte Carlo method (28) is used at fixed test-period for Z-statistics calculation, for which a way of choosing random elements for the matrixes is not pointed out. Furthermore, at long test-periods (∼50 bp and more) for reliability of Monte Carlo results, a number of the tests that are needed is too large (even for powerful computer). Again, in general case, when there is little number of the repeats for test-period, initial matrix, used in Z-statistics calculations, becomes statistically instable. So, in general, for long test-periods or for a small number of their repeats, usage of Z-statistics becomes problematic.

In the earlier works (33, 34), the methods for recognizing new type of latent periodicity, called latent profile periodicity (latent profility), have been elaborated. This type of latent periodicity expands on the notion of approximate tandem repeat. It appears that the HeteroGenome database has collected many DNA sequences in which latent profile periodicity is recognized. To compare a possibility of revealing the latent periodicity with the help of web-server LEPSCAN (30), a number of DNA sequences, in which such profile periodicity with period of <20 bp had been recognized, were selected according to the upper period border allowed for search by the LEPSCAN. No latent periodicity was found by this web-server in the selected sequences. Let us give a particular example of the two such DNA sequences from the HeteroGenome. These sequences were from *A. thaliana* chromosome I (11780 828–11 780  960 bp and 11 983 025–11 983 293 bp). In the first sequence, the latent profile periodicity with period of the 4 bp has been recognized and in the second one, with period of the 11 bp.

## Results

Applying the SS-approach (26, 32) enabled us to develop a complex program that was able to reliably reveal latent periodicity regions of differing types (approximate tandem repeats, fuzzy repeats, profile periodicity). The level of pattern copy divergence in tandem repeats was limited to 50%. The program revealed equally well microsatellites with patterns in the range of 2–10 bp and minisatellites with patterns ∼10–100 bp, as well as megasatellites with longer patterns up to 2000 bp. The minimum copy number in the revealed approximate tandem repeats and fuzzy repeats was two.

Along with highly divergent tandem repeats, regions of the new type of latent periodicity known as latent profile periodicity (33, 34) were also revealed.

Reliable (at significance level α = 10^−6^) heterogeneity regions, mainly approximate tandem repeats, revealed in the entire genomes of *S. cerevisiae*, *A. thaliana*, *C. elegans* and *D. melanogaster* were collected in the HeteroGenome database (www.jcbi.ru/lp_baze).

### Description of the database

**Figure 5.**
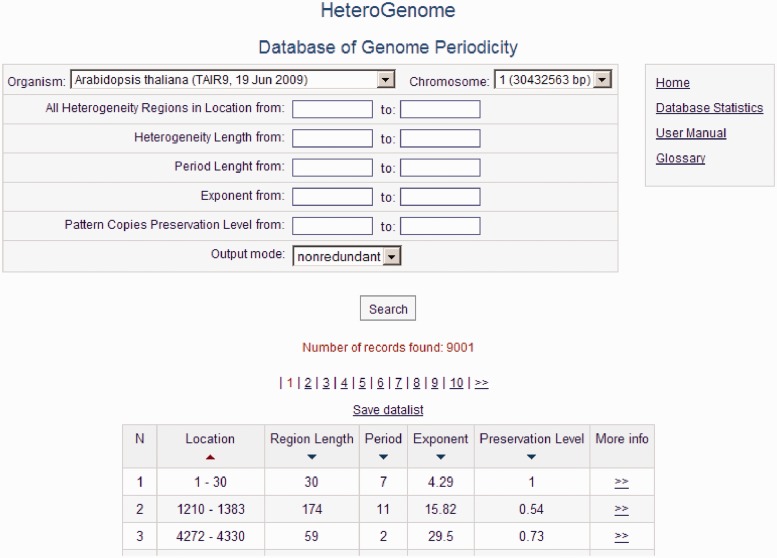
Query form and data output. Output is shown of all reliable heterogeneity (latent periodicity) regions on chromosome I of *A. thaliana* corresponding to the first, nonredundant, level of the HeteroGenome records.

**Figure 6.**
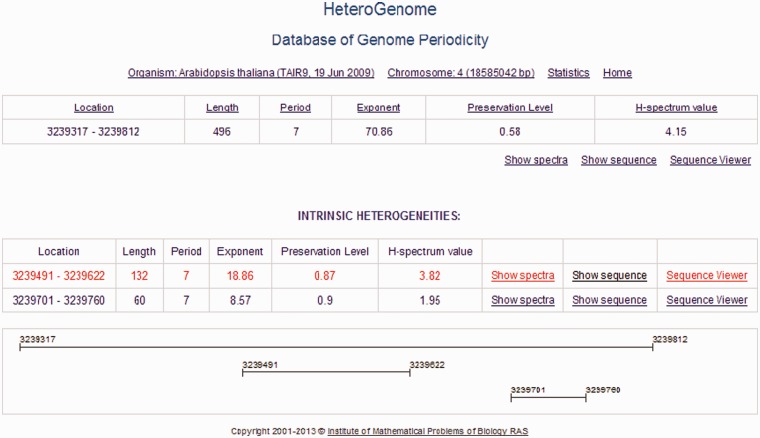
Two-level structure of a record in HeteroGenome. DNA sequences with the revealed latent periodicity of 7 bp on chromosome IV of *A. thaliana* constitute a group in which the two levels of structure are distinguished. A sequence of the greatest length, representing the entire group, is placed at the first level. The remaining sequences making up the parts of the representative sequence correspond to internal representative regions of clear periodicity structure and form the second level (INTRINSIC HETEROGENEITIES) in the group. A graphical schema of the group is shown at the bottom. The sequences at the first level from all groups constitute nonredundant data in HeteroGenome.

HeteroGenome is relational database managed by MySQL. For user convenience, the data search is organized across all fields of the database (see Figure 5) with the option of sorting data by field (Location, Region Length, Period, Exponent, Preservation Level). A detailed description of all the fields and their acceptable values is given in separate windows accessible by clicking on the fields’ names. The User manual with the examples demonstrating how to work with the database is placed on the site. Search results can be downloaded as a plain text file. Because of the two-level structure of each record (see Figure 6), described in the next section, the database interface offers two modes of information query: nonredundant, in which the sequences of the first level are searched; and simple, in which all sequences in the database are searched.

**Figure 7.**
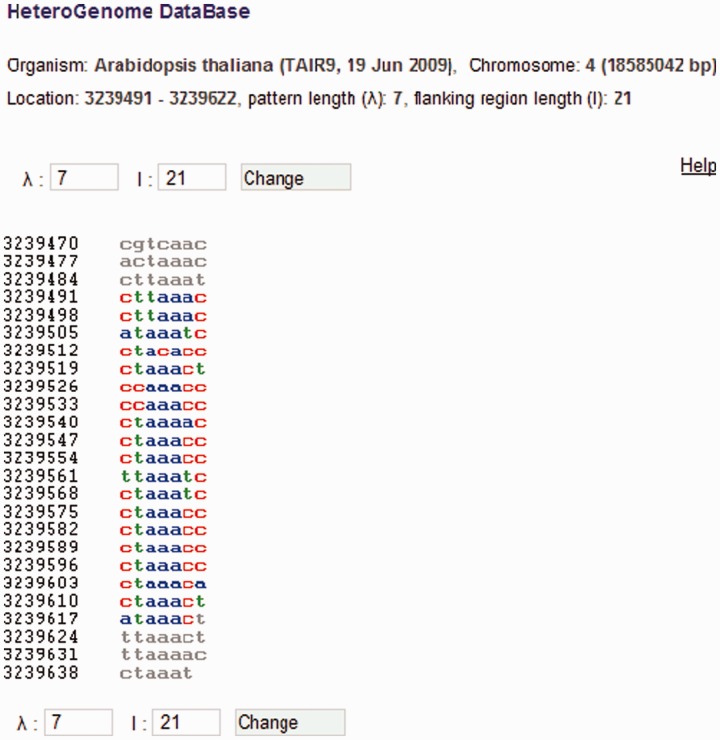
DNA sequence view in HeteroGenome. Consecutive segmentation of a DNA sequence on chromosome IV (3239491–3239622 bp) of *A. thaliana* is shown corresponding to the revealed latent periodicity pattern size *λ* = 7 bp (see Figure 6, the sequence parameters are shown in red). Left and right flanking regions of length *l* = 21 bp are also shown. Segment size *λ* and length *l* of flanking region can be redefined. Pressing the ‘Change’ button generates corresponding sequence segmentation in a new window.

For each sequence in HeteroGenome, separate windows show the user: (i) **H**- and **pl**-spectrum [see Equations (4) and (5) and Figure 1], based on which an estimate of pattern periodicity size is proposed; and (ii) the analyzed DNA sequence presented as a column of consecutive sequence segments with lengths equal to the pattern size estimate (see Figure 7). With information obtained from the spectral visual analysis, the user has the option of changing the segment length to more exactly estimate the pattern size for the approximate tandem repeat. Moreover, by following a link to the Sequence Viewer (http://www.ncbi.nlm.nih.gov/projects/sviewer/) graphical interface, the user is able to obtain information on the functional context of chromosomal location data.

#### Two levels of data presentation

For the presentation of nonredundant data in HeteroGenome, we developed a logical unit record as a group of DNA sequences with reliable heterogeneity (latent periodicity), which intersect or, as a rule, have the same or multiple period lengths. Two levels of data presentation were distinguished in the group. At the first level, the longest DNA sequence, which is representative of the group, is considered. The remaining sequences in the group belong to the second level. Generally, these are well-determined local periodicity fragments of the group representative. An example of such a record, showing the two-level organization, can be seen in Figure 6.

In addition to the groups containing DNA sequences of both levels (the group representative and elements of its internal heterogeneity) in HeteroGenome, other groups contain only a single DNA sequence representative of the group. Individual groups do not intersect. Thus, sequences representing the groups form nonredundant chromosome coverage by regions of reliable heterogeneity (latent periodicity).

HeteroGenome can be searched for certain information on latent periodicity (with a given period length, periodicity region size, character preservation level **pl** for pattern copies and so on), as described in the following sections, at the first (nonredundant) level only, or at the first and second levels together.

#### Additional sequence analysis

Period length and coordinates for any region of latent periodicity can be determined in HeteroGenome with greater accuracy by using the results of visual analysis of the spectral parameters (see Figure 1), by subdividing the sequence into segments of estimated period length (see Figure 7), and by providing a length for flanking region sequences. An example of such analysis is described in the User manual on the database site.

Additional analysis can lead to a more precise reading of the data because the data specification and data distribution across the groups are generated automatically. After studying group content, the user can correct the group coordinates or even consider the group as consisting of several subgroups.

### Analysis of latent periodicity in HeteroGenome

A comparison of data for the genomes of *S. cerevisiae*, *A. thaliana*, *C. elegans* and *D. melanogaster* in HeteroGenome and TRDB (24) shows that HeteroGenome in fact contains all the repeats presented in TRDB and supplements them with data on highly divergent tandem repeats.

**Figure 8.**
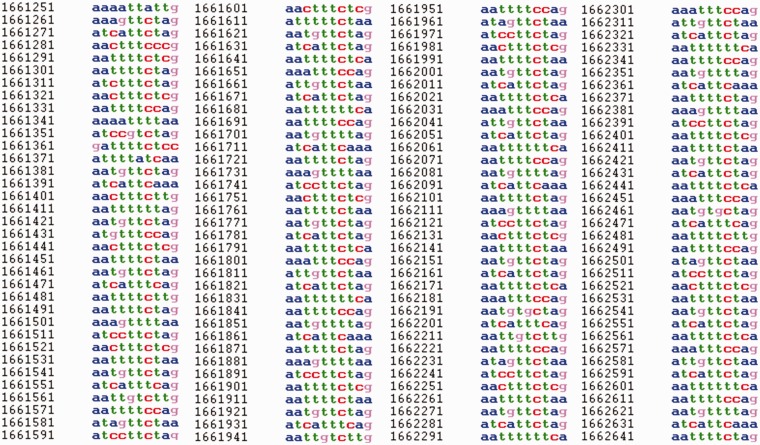
Fragment of a sequence on chromosome I (1 661 021–1 663 249 bp) of *C. elegans* in HeteroGenome for which a latent profility of 10 bp was revealed. For displayed fragment **pl**(10) = 0.77

HeteroGenome also indicates regions of the new type of periodicity called latent profile periodicity or profility (33, 34) in the genome. The notion of latent profility expands on the notion of the tandem repeat. A pattern of latent profility consists of independent random characters with a corresponding probability distribution for letters of the DNA alphabet. This type of periodicity requires future research. Figure 8 shows an example of a sequence with a profility of period length 10 bp, obtained from HeteroGenome.

It is interesting to note that TRDB (24) contains ∼40 different tandem repeats for the fragment shown in Figure 8, but only one of them has a pattern size of 10 bp (5.4 pattern copies). Nevertheless, there is a clear structural regularity of 10 bp for the fragment. The presence of a latent profile periodicity of 10 bp in the sequence has been verified by the methods described in (33, 34).

#### Genome coverage by latent periodicity

An important quantitative index used to study the evolution and functional meaning of latent periodicity regions in genomes is the amount of genome coverage by these regions. Nonredundant data on regions of reliable heterogeneity (latent periodicity) enables a sufficiently precise estimation of the percentage of latent periodicity regions (including highly divergent tandem repeats and profility) in analyzed genomes. Table 2 presents these estimates.

**Table 2.** Proportion of regions with reliable heterogeneity (latent periodicity) in analyzed genomes

Species	Genome length, bp	Total heterogeneity length, bp	Percentage of heterogeneity
*S. cerevisiae* MT	85 779	20 892	24.4
*S. cerevisiae*	12 070 900	419 909	3.5
*A. thaliana*	119 146 348	4 247 672	3.6
*C. elegans*	100 269 917	6 692 629	6.7
*D. melanogaster*	120 381 546	5 108 483	4.2

**Figure 9.**
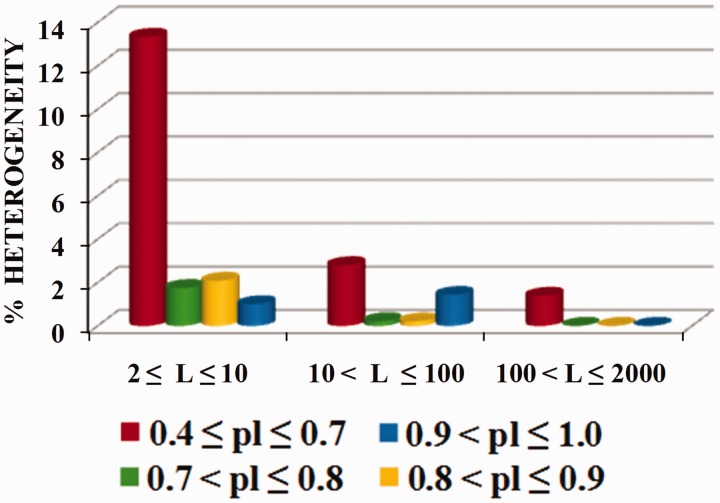
Histogram of structural content for latent periodicity regions in mitochondrial genome of *S. cerevisiae*. Corresponding to revealed period *L*, for micro- (2 ≤ *L* ≤ 10), mini- (10 < *L* ≤ 100) and megasatellites (100 < *L* ≤ 2000), coverage (as a percentage) of the mitochondrial genome by latent periodicity regions with various preservation levels [**pl**(*L*), see Equation (5)] are shown separately. The preservation level in range 0.4 ≤ **pl** ≤ 0.7 (red) corresponds to highly divergent tandem repeats; that in range 0.7 < **pl** ≤ 0.8 (green) corresponds to moderately divergent tandem repeats; that in 0.8 < **pl** ≤ 0.9 (yellow) corresponds to slightly divergent tandem repeats; and that in 0.9 < **pl** ≤ 1.0 (blue) corresponds to perfect tandem repeats.

A high percentage (24.4%) of latent periodicity regions was revealed in the yeast *S. cerevisiae* mitochondrial genome. As Figure 9 indicates, more than half of the regions (13.3%) are highly divergent microsatellites.

The majority of latent periodicity regions in the nuclear genomes of the analyzed organisms consist of micro- and minisatellites (period length <100 bp). In the human genome, the proportion of micro- and minisatellites is commonly estimated to be 3% (36). Together with those repeats with periods that do not exceed 2000 bp, the proportion of tandem repeats is ∼10% (19). Taking into account the data in Table 2, we may suppose that the periodicity, shown by tandem repeats with periods <2000 bp, varies in eukaryotic genomes to within 10%. This percentage may be conditioned by a balance between a molecular mechanism for the origin of tandem repeats and their divergence, which thus stabilizes the repeat length.

#### Latent periodicity impact on chromosome length

**Figure 10.**
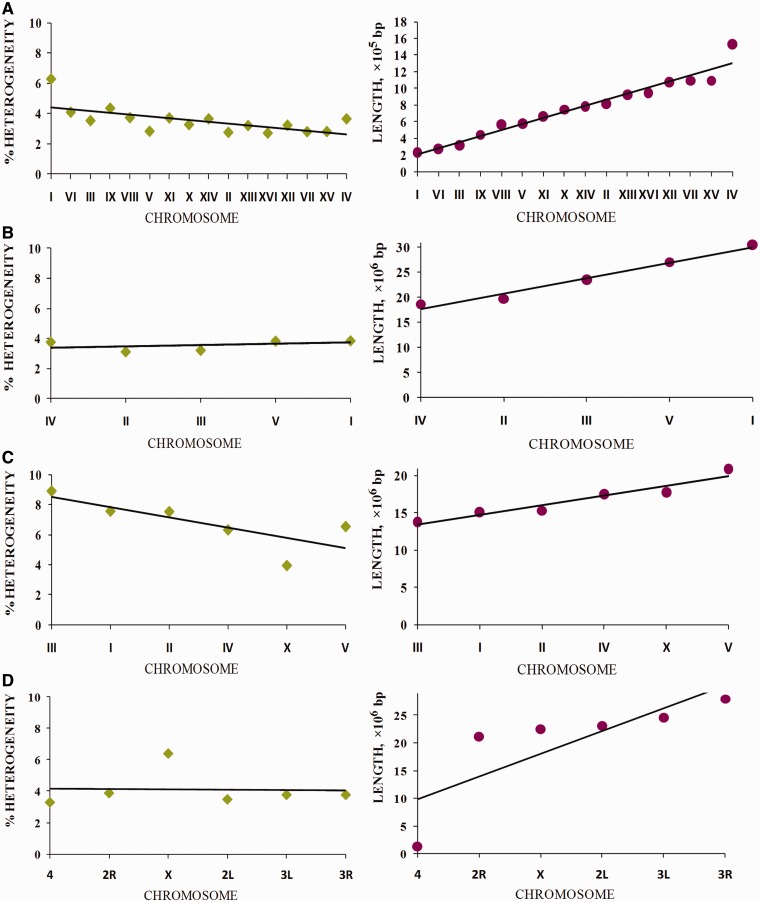
Coverage of chromosomes of analyzed organisms by regions of reliable heterogeneity (latent periodicity). For each organism, *S. cerevisiae* (**A**), *A. thaliana* (**B**), *C. elegans* (**C**) and *D. melanogaster* (**D**), chromosomes are ordered by ascending length, as shown in the respective images on the right. Solid straight lines show trends. Percentage of latent periodicity regions on each chromosome is determined at the nonredundant level of records in HeteroGenome. See the text and Figure 6 for details.

Let us consider how the proportion of latent periodicity regions (reliable heterogeneity regions shown by tandem repeats) depends on the length of the chromosomes of the analyzed organisms (see Figure 10). Specific dispersion as a percentage of latent periodicity regions covering single chromosomes was observed for each model organism. The highest dispersion (4.95%) between maximal (8.91% on chromosome I) and minimal (3.96% on chromosome X) coverage was observed in the *C. elegans* genome. In the genome of *D. melanogaster*, this dispersion is approximately one-third less (3.11%), ranging from 3.30% (chromosome IV) to 6.41% (chromosome X). For both organisms, the amount of dispersion is caused by the particular location of the X chromosome. Moreover, in the case of *C. elegans*, the chromosome has minimal coverage by percentage, but in *D. melanogaster* the X chromosome has the most coverage of all chromosomes in the genome.

Dispersion of chromosome coverage by latent periodicity regions for the *D. melanogaster* genome is comparable with that for *S. cerevisiae*. Dispersion for *S. cerevisiae* (3.57%) ranges from 2.7% (chromosome XVI) to 6.27% (chromosome I). Note that chromosome I, which has the highest periodicity percentage in the *S. cerevisiae* genome, is the shortest chromosome of the yeast.

Although in the genomes of *S. cerevisiae*, *C. elegans* and *D. melanogaster*, percentage of latent periodicity dispersion on chromosomes is comparable with the average percentage of periodicity in the single genomes, in the genome of *A. thaliana* this dispersion does not exceed 0.75%. As we can see in Figure 10B for arabidopsis, with growth in chromosome length, the proportion of latent periodicity regions remains almost constant.

In fact, for all analyzed genomes of the model organisms, with increasing chromosome length the proportion of latent periodicity regions is generally fixed or decreases. We may suppose that, because of their instability and ability to elongate during DNA replication, the tandem repeats moderately (∼10%) influenced the length of chromosomes of the model organisms.

The most probable mechanism repressing the elongation of periodicity regions is a divergence that is sufficiently rapid and prevents slippage of DNA strings during chromosome replication.

#### Structure preservation in latent periodicity regions

**Figure 11.**
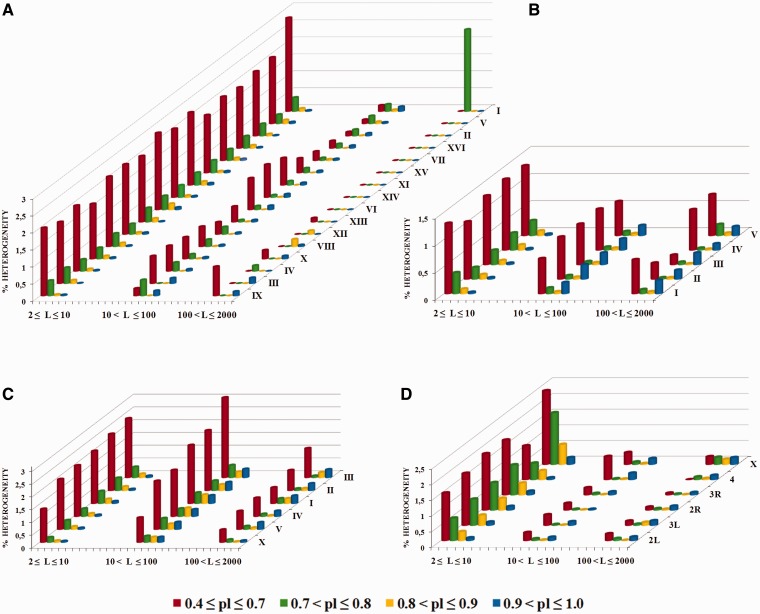
Structural content for latent periodicity regions in the genomes of *S. cerevisiae* (**A**), *A. thaliana* (**B**), *C. elegans* (**C**) and *D. melanogaster* (**D**). Corresponding to revealed period *L*, for micro- (2 ≤ *L* ≤ 10), mini- (10 < *L* ≤ 100) and megasatellites (100 < *L* ≤ 2000), coverage (as a percentage) of genome by periodicity regions with various preservation levels [**pl**(*L*), see Equation (5)] are shown as separate histograms. The preservation level in range 0.4 ≤ **pl** ≤ 0.7 (red) corresponds to highly divergent tandem repeats; that in range 0.7 < **pl** ≤ 0.8 (green) corresponds to moderately divergent tandem repeats; that in 0.8 < **pl** ≤ 0.9 (yellow) corresponds to slightly divergent tandem repeats; and that in 0.9 < **pl** ≤ 1.0 (blue) corresponds to perfect tandem repeats. The order of chromosomes in the figure was determined by visual similarities between their histograms

Figure 11 shows histograms of the qualitative structural content of the revealed periodicity regions for all chromosomes in the genomes of the model organisms. Percentages of chromosome length occupied by highly divergent, moderately divergent, slightly divergent and perfect tandem repeats are shown separately for the micro-, mini- and megasatellites.

As can be seen from Figure 11A, latent periodicity regions in the *S. cerevisiae* genome are generally indicated by highly divergent sequences of microsatellites, which make up ∼2% of regions in the genome. Highly divergent minisatellites make up <1% of the genome. Except for chromosomes I and IX, megasatellite repeats with period lengths >100 bp are absent in the yeast genome.

Some chromosomes in the genome of *S. cerevisiae* have similar histograms for all three considered groups of satellites, such as chromosomes VIII and XII, chromosomes XIII, VI and XIV and chromosomes XI, XV and VII.

For the plant *A. thaliana* (Figure 11B) and the nematode *C. elegans* (Figure 11C), the genome length of which has a greater order of magnitude than that of the yeast *S. cerevisiae* (Figure 11A), the histograms show similar tendencies in terms of the qualitative makeup of the tandem repeats in the genomes.

Firstly, in the genomes of *A. thaliana* and *C. elegans*, highly divergent minisatellites account for ∼1–1.5%, which is a perceptible proportion and is similar to their percentages of microsatellites. Therefore, in *A. thaliana* and *C. elegans*, mini- and microsatellites contribute similarly to structural and functional genome organization. Secondly, some megasatellite repeats, which are almost completely absent in the yeast genome and, as described below, are numerically insignificant in the *Drosophila* genome (Figure 11D), in the genomes of arabidopsis and nematode are sufficiently important and make up ∼1% of regions.

Neglecting some particularities for chromosomes II and III, the histograms for *A. thaliana* chromosomes are similar (see Figure 11B). In fact, except for the chromosome X and III histograms, the remaining histograms for *C. elegans* (see Figure 11C) are identical.

Histograms produced using the results of structural analysis of latent periodicity regions revealed on the chromosomes of fruit fly *D. melanogaster* are shown in Figure 11D. It can be seen that highly divergent (∼1.5–2%) and moderately divergent microsatellites (∼0.5–1%) are dominant in the *Drosophila* genome. Note the similarities between histograms in Figure 11D, with the exception of those for chromosomes 4 and X.

Similarities between histograms reflecting the qualitative structural content of latent periodicity regions on the single chromosomes of the analyzed organisms are not evidence of a common evolutionary origin of the chromosomes; however, they do allow us to propose a similar mechanism for the evolutionary pressure and divergence under which the chromosomes were formed.

Moreover, as indicated in Figure 11, in each genome we can differentiate one or two dominant characteristic types of latent periodicity such as highly divergent microsatellites in the *S. cerevisiae* genome. The genomes of *A. thaliana* and *C. elegans* have similar percentages of characteristic types of latent periodicity (highly divergent micro- and minisatellites ∼1.5%).

Determining the functional role of dominant periodicities in a genome is likely to be a topic of future research.

#### Latent periodicity in functional regions

Via a hyperlink to the Sequence Viewer graphical interface (http://www.ncbi.nlm.nih.gov/projects/sviewer/), for any DNA region in HeteroGenome, information can be obtained about the region intersection with annotated sequences of the investigated genome. Table 3 presents a general overview of HeteroGenome data distribution over functional (annotated) and functionally unassigned, usually noncoding, DNA sequences of genomes.

**Table 3.** HeteroGenome groups’ distribution over functional regions in the genomes

GenBank features	*S. cerevisiae**^a^*	*A. thaliana*	*C. elegans*	*D. melanogaster*
Gene	3276	21598	25551	48657
mRNA	–	15337	12667	23879
CDS	3269	13370	12046	19463
Intron^b^	6	3105	14145	28112
Exon	–	–	–	21
STS	–	141	–	150
tRNA	–	5	6	2
rRNA	–	2	–	8
ncRNA	–	3	–	–
misc_RNA	4	–	–	–
rep_origin	15	–	–	–
repeat_region	95	–	–	1951
LTR	11	–	–	–
Unassigned	738	12 935	13 773	22 851
HeteroGenome Groups	4094	34 566	39 329	72 772

^a^There are no mRNA features in the annotation for *S. cerevisiae* genome, ^b^The data on the introns were obtained from attribute line ‘join’ for ‘mRNA’ (‘CDS’ for *S. cerevisiae*) feature description.

To estimate the distribution of the HeteroGenome groups over annotated regions of genome, a criterion that is nonstrict enough was used. If the intersection area between group and functional region was not less 50% of minor sequences, then the group is assigned to considered region. While estimating the groups’ distribution over annotated regions, an alternative splicing was not taken into account, i.e. for one gene only one mRNA and Coding DNA Sequence (CDS) were considered.

As one can see from the Table 3, according to the criterion introduced above, for the genomes of *S. cerevisiae*, *A. thaliana*, *C. elegans* and *D. melanogaster*, correspondingly, 80, 62, 65 and 67% of the HeteroGenome groups are located in the genes. In accordance with the list of organisms, 18, 37.4, 35 and 31.4% of the groups are located in unannotated (unassigned) regions of their genomes. In general, distribution over other functional regions, besides the genes, is random and unessential. However, it should be mentioned that 2.6% of the HeteroGenome’s groups are located in various repeats of *D. melanogaster*.

For the groups, located in the genes, there is a high probability to be assigned both to intron and exon because many groups cross the boarders between them. So, additional analysis was done to calculate the sums of the nucleotides (in the HeteroGenome groups) occurring in the introns and exons separately. Table 4 presents the results of such an analysis, in relation with total number of the nucleotides occurring in functional regions of whole genome. Comparing Tables 2 and 4, one can see that the shares of nucleotides, located in the genes, for all organisms in the HeteroGenome are practically similar to the shares of genome coverage by the regions of latent periodicity. The distribution of nucleotides over exons and introns depends on the organism. Thus, for example, the percentage of the whole length of latent periodicity regions in the exons of *D. melanogaster* (12%) is higher than in the introns (9%), despite the fact that the introns are nearly twice longer than the exons. On the contrary, with whole length of the exons and introns being similar in *C. elegans* genome, share of latent periodicity regions in the introns is twice higher than it is in the exons (10 and 5.4%, correspondingly). Hence, direct dependence is not observed between the whole length of latent periodicity regions and genome length, or between functional regions’ total length.

**Table 4.** The whole number of the nucleotides from the HeteroGenome groups over gene regions in the genomes

Species	Nucleotides in the groups**/**nucleotides in genome		
	Genes	Exons^a^	Introns^a^
*S. cerevisiae*	354 459**/**8 829 668, (4%)	352 781**/**8 737 430, (4%)	448**/**64 756, (0.7%)
*A. thaliana*	2 037 728**/**70 751 773, (3%)	1 601 059**/**40 167 753, (4%)	296 614**/**19 355 644, (1.5%)
*C. elegans*	3 816 463**/**60 377 579, (6.3%)	1 479 515**/**27 267 246, (5.4%)	402 554**/**32 373 603, (10%)
*D. melanogaster*	3 786 123**/**79 344 223, (4.8%)	3 410 523**/**28 271 547, (12%)	4 419 548**/**49 121 309, (9%)

*Note*: The share of groups’ nucleotides in the total length of corresponding functional regions in genome is shown in parenthesis.

^a^The data on the exons and introns were obtained from attribute line ‘join’ for ‘mRNA’ feature description (‘CDS’ feature description for *S. cerevisiae*).

#### Density distribution of latent periodicity regions over the chromosomes

**Figure 12.**
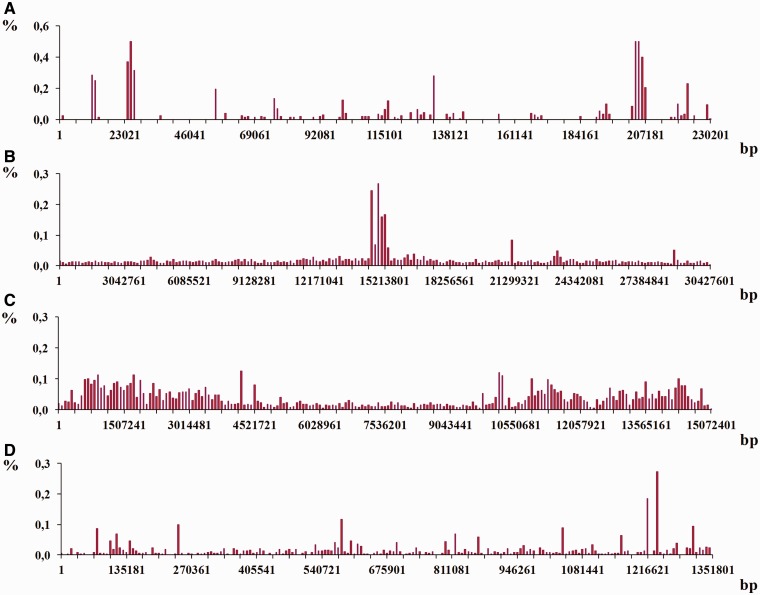
An example of the histograms showing density distributions for latent periodicity regions revealed on the chromosomes. The height of histogram bar equals a part of the whole percentage of latent periodicity regions on chromosome, associated with every sequential fragment of length equal to histogram’s step (see text for details). (**A–C**) Histograms show the distributions for the chromosomes I from the genomes *S. cerevisiae*, *A. thaliana* and *C. elegans*, correspondingly. (**D**) Histogram is shown for chromosome IV from the genome of *D. melanogaster*.

The study of density distribution of latent periodicity regions over the chromosomes was done for all analyzed genomes in the work. Each chromosome was divided into consecutive fragments of the same size that account for 0.5% of the whole chromosome length. Such a size is considered an interval of division. Then a sum of the lengths (in nucleotides) for latent periodicity regions located within the fragment’s boarders was calculated for each fragment. Such a sum, normed at the length of the whole chromosome and multiplied by 100%, was considered a part from the whole percentage of latent periodicity regions on chromosome, which was assigned to the each fragment. Summarizing all parts in the division gives an estimate of general percentage of latent periodicity on chromosome. Distributions of numerical values for such shares over the four chromosomes from the organisms considered in the work are shown in Figure 12. In investigating density distribution of latent periodicity regions, only the sequences that represent the groups were taken into account as giving nonredundant estimate of chromosome coverage by latent periodicity.

Histograms in Figure 12 demonstrate the density distribution for the regions of latent periodicity over chromosomes I from the genomes of yeast *S. cerevisiae* (A), plant *A. thaliana* (B) and nematode *C. elegans* (С), and also over chromosome 4 from the genome of fruit fly *D. melanogaster* (D). Corresponding steps of the divisions for the chromosomes are equal to 1151, 152 138, 75 362 and 6759 bp. Results for all chromosomes of considered organisms are present on page Statistics of the HeteroGenome database (http://www.jcbi.ru/lp_baze/).

**Figure 13.**
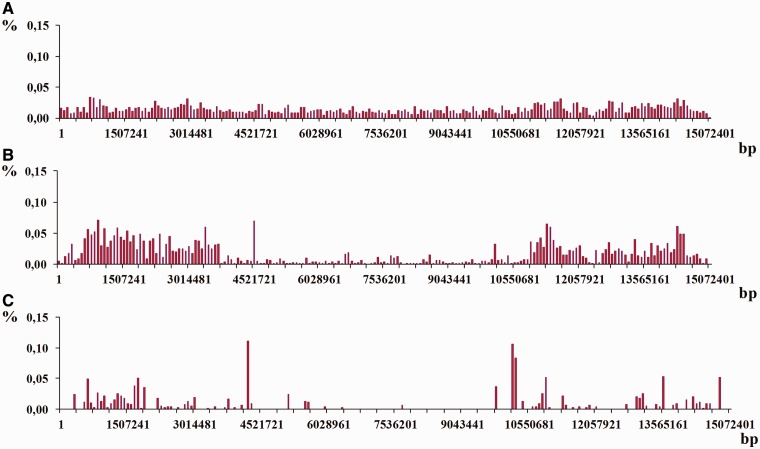
Histograms of density distribution for the regions of latent periodicity revealed on chromosome I of *C. elegans* genome are shown for the three classes of periodicity—(**A**) micro-, (**B**) mini- and (**C**) megasaellites.

Moreover, for each chromosome the three additional density distributions were obtained following three different classes of periodicity in genome, i.e. micro-, mini- and megasatellites with period lengths in the intervals 2 ≤ *L* ≤ 10, 10 < *L* ≤ 100 and 100 < *L* ≤ 2000, correspondingly. Figure 13 shows an example of such distributions for chromosome I from *C. elegans* genome (see Figure 12C) for their combined distribution).

As it was demonstrated in the examples in Figures 12 and 13, density distribution of the latent periodicity over the chromosomes unambiguously characterizes each chromosome in the genomes. Such distribution may be considered as some kind of DNA fingerprint or bar code for every chromosome in the genomes of various organisms.

## Conclusions

As a result of applying the SS-approach to revealing heterogeneity (latent periodicity) regions in the genomes of model organisms—*S. cerevisiae*, *A. thaliana*, *C. elegans* and *D. melanogaster*—reliable data were obtained for tandem repeats, including highly divergent repeats and regions of a new type of latent periodicity called profility (33, 34) (see Figure 8 for example), from the HeteroGenome database.

Because of its user-friendly interface and options for additional data analysis, HeteroGenome may be useful both in searching for new markers in molecular genetic investigations of organisms and for advanced research into latent periodicity in DNA sequences. The first efforts to research latent periodicity phenomenon in genome with the help of HeteroGenome database have been done in (38).

Latent profility may be located with the aid of the methods described in (33, 34) for a number of regions where 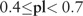 [see Equation (5) and Figure 1]. A specially developed two-level structure of records in HeteroGenome enables the data to be presented without redundancy and indicates conservative fragments in the regions of latent periodicity.

Taking into consideration the HeteroGenome data for the genomes of *S. cerevisiae*, *A. thaliana*, *C. elegans* and *D. melanogaster*, and the results of other research (19, 36), it may be that latent periodicity regions constitute ∼10% of regions in the genomes of various organisms. Highly divergent microsatellite repeats (with period lengths <10 bp), amounting to ∼2% of the entire genome length are dominant in all of the aforementioned organisms. As we have described, the qualitative and quantitative content of regions of latent periodicity are characteristic of the genomes. For example (see Figure 11), in the genome of yeast *S. cerevisiae* (except for chromosomes I and IX) and in that of fruit fly *D. melanogaster*, megasatellite repeats (with period lengths >100 bp) are almost completely absent. In contrast, megasatellite repeats in the genome of plant *A. thaliana* and in that of nematode *C. elegans* are sufficiently noticeable and account for ∼1% of the genomes.

Analysis of the HeteroGenome data distribution over functional (annotated in GenBank) and nonfunctional (unassigned) genome DNA sequences shows that the share of latent periodicity in the genes is similar to the general genome coverage by the regions of latent periodicity. Nevertheless the regions’ distribution between the exons and the introns depends on the organism. Thus, a percentage of the whole length of latent periodicity regions in the exons of *D. melanogaster* (12%) is higher than in the introns (9%), though the introns’ length is nearly twice greater the exons’ length. On the contrary, while the whole length of exons and introns are similar in *C. elegans* genome, the share of latent periodicity regions in the introns is twice higher than it is in the exons (10 and 5.4%, correspondingly).Therefore, the direct dependence is not observed between the length of latent periodicity regions and genome or its functional regions total length.

It should be noted that general distribution of latent periodicity regions over the chromosomes in genome is a unique characteristic for each of the considered model organisms.

## Funding

Russian Foundation for Fundamental Research (12-07-00530).

*Conflict of interest.* None declared.
